# A Two-Week Vacation in the Tropics and Psychological Well-Being—An Observational Follow-Up Study

**DOI:** 10.3390/ijerph191610381

**Published:** 2022-08-20

**Authors:** Tanja Laukkala, Tom Rosenström, Anu Kantele

**Affiliations:** 1 Department of Psychiatry, University of Helsinki and Acute Psychiatry and Consultations, HUS Helsinki University Hospital, 00029 Helsinki, Finland; 2 Department of Psychology and Logopedics, Faculty of Medicine, University of Helsinki, 00014 Helsinki, Finland; 3 Meilahti Infectious Diseases and Vaccine Research Center, MeiVac, Department of Infectious Diseases, Inflammatory Center, University of Helsinki and Helsinki University Hospital, 00029 Helsinki, Finland

**Keywords:** tropical holiday, travelers’ diarrhoea, psychological well-being, psychological distress

## Abstract

Despite the vast annual number of international visitors to the tropics, surprisingly little data are available on the psychological well-being associated with the travels or with travelers’ diarrhoea (TD). We herein recruited participants of a vaccination trial, OEV-123, before their 12-day holiday in Benin, West Africa. We assessed the travelers’ psychological distress with a general health questionnaire (GHQ-12) and retrieved data on TD from the trial database. The GHQ-12 was completed before (wave 0), at return (wave 1), and 1-month after (wave 2) the trip. Of the 174 participants, 73% were women, with a mean age 40 years. Moreover, 24% reported psychological distress before traveling, 10% immediately after, and 16% 1-month after the trip (GHQ-12, 3 or more; 0–12 scoring). The findings showed that psychological well-being increased after the tropical holiday. The GHQ-12 middle wave sum score differed from the wave 0 (*p* < 0.001) and wave 2 (*p* = 0.008) sum scores, with travelers reporting highest levels of well-being on their return, with evidence of a lasting improvement. TD was experienced by 71%, and it had a negative impact on psychological well-being only if experienced after travel.

## 1. Introduction

The annual number of travelers to the tropics exceeds 300 million, according to the World Tourism Organization (UNWTO; https://www.unwto.org/statistic/basic-tourism-statistics). The majority of international arrivals in emerging market economies are for holidays. The number of arrivals has been increasing every year until the current SARS-CoV-2 pandemic; in 2019 it reached 685 million. Holidays’ positive effect on mental well-being is an intuitive explanation for why people travel to tropics so much despite the behaviour´s obvious financial and environmental costs, and frequent costs in terms of physical health, such as contracting travelers’ diarrhoea (TD). However, scientific evidence based on this hypothesis remains limited. Despite these vast numbers of leisure-time travelers, research data on the impact of these journeys to the tropics on the psychological distress or well-being of the travelers are surprisingly scarce [1,2,3,4]. As an exception, winter depression, a seasonal affective disorder subtype has been studied more extensively. Sufferers of winter depression have been found to respond to light therapy [5], received as artificial light or sunshine. In fact, light therapy may play a role both in the prevention and in the treatment of winter depression.

Some of the few other published studies on travel and studies on well-being suggest meditation and holistic wellness programs as a means of prolonging the benefits of a vacation [2,6]. Warmer and sunnier vacation locations are expected to facilitate recuperation [7]. Similarly, vacations away from home have been considered psychologically beneficial, although these effects can be decreased by simultaneous work stress and a consequent inability to relax [8].

The aim of this study was to assess the impact of tropical holidays on psychological well-being. Participants of a clinical randomized controlled vaccination trial for ETVAX^®^, an oral diarrhoeal vaccine against Enterotoxigenic Escherichia coli, were also recruited into this study of psychological well-being. They all traveled to the tropics from a Nordic country, wherein winters offer little light in general, and in some days, sun does not even rise at all at the northern regions. We hypothesized that a vacation-like trip temporarily increases psychological well-being among healthy participants and that the effect at least partly diminishes during follow-up. Moreover, we wanted to explore whether the time of year and contracting TD during or after travel affected the travelers’ psychological well-being. To the best of our knowledge, this is the first study on travel-related well-being using the validated GHQ-12 questionnaire aimed at evaluating psychological well-being [9].

## 2. Materials and Methods

### 2.1. Study Design

The present study was designed to explore psychological well-being among travelers to the tropics. Volunteers were recruited among the participants of a clinical field trial OEV-123 for ETVAX^®^, (Scandinavian Biopharma, Solna, Sweden) an oral diarrhoeal vaccine against Enterotoxigenic Escherichia coli (ETEC). All participants of the OEV-123 trial went on a 12-day trip to Benin, West Africa. As the OEV-123 trial was designed to have only a minor impact on the course of the holiday, it provided us with an opportunity to explore the psychological well-being of travelers taking a holiday in the tropics. Participants of OEV-vaccination trial were generally healthy adults, who traveled to a tropical holiday. The present study was a prospective study conducted from June 2018 to March 2019 using questionnaires collected before, during, and after travel.

This observational study protocol was approved by the Ethics Committee of the Helsinki University Hospital (HUS/1595/2017) and permission to conduct the study was provided by the Helsinki University Hospital (HUS/64/2018). This observational study was conducted according to the Declaration of Helsinki. All participants provided their written informed consent.

### 2.2. Questionnaires

We used General Health Questionnaire-12, which is a screening instrument for psychological distress with 12 questions, an instrument also widely used in epidemiological studies (GHQ-12) [10,11]. Questions assess psychological distress and the ability to function, e.g., whether the respondent felt unhappy and depressed, felt capable of making decisions about things, and has been thinking of him/or herself as a worthless person. In addition, Finnish translation of GHQ-12 was used. All GHQ-12 items have a 4-point scoring system, ranging from a “better/healthier than normal” option, to “same as usual”, “worse/more than usual’ or “much worse/more than usual”. The resulting full sum score range is from 0 indicating no psychological distress to 36 indicating maximum experienced psychological distress, but the items are often re-scored using a 0-0-1-1-scoring; “better” and “usual” responses are scored as 0, and “worse” and “much worse” responses are scored as 1. The responses to individual re-scored items are summed to yield a total score varying from 0 to 12, for which 3 or more points were considered to refer to psychological distress in earlier assessments of the general Finnish population [12,13,14]. GHQ-12 is considered to have reasonably good psychometric properties, and the Finnish translation has been used earlier in population-based samples [12,13,14]. In this observational study, GHQ-12 was used to assess psychological well-being before, immediately after, and 1-month after the vacation-like trip to Gran Popo, Benin in West Africa. As a main additional inconvinience compared to a normal vacation, the randomized clinical trial (RCT), participants agreed to inform the OEV-123 trial personnel of TD symptoms and to stay overnight in a pre-determined hotel to enable the collection of stool samples in the case of diarrhoea.

The analysis included GHQ-12 questionnaires before the holiday, which was part of the vaccination field trial, as well as immediately, and 1-month after the trip. Moreover, the time points were selected to be feasible in relation to vaccination RCT contact points. Altogether, 174 of the ETVAX participants between June 2018 and March 2019 chose to participate in our study, with the majority being women (73%) and 27% were men. The age (mean) of the participants was 40 years in the first questionnaire. The baseline measurement (before trip) is referred to as “wave 0”, the middle measurement (after trip) as “wave 1”, and the last (1-month follow-up) measurement as “wave 2”.

### 2.3. OEV-123 Field Trial

OEV-123 was a randomized, placebo-controlled study exploring, among others, the safety, immunogenicity, and efficacy of ETVAX, the most advanced vaccine against Enterotoxigenic Escherichia coli (ETEC), an agent that causes diarrhoea, stunting, and childhood mortality in developing countries. Moreover, ETEC causes travelers’ diarrhoea in travelers visiting tropical regions. OEV-123 tested ETVAX against a placebo in a double-blinded study setting among 729 healthy Finnish travelers who all traveled to Benin, West Africa and provided stool samples before, during, and after travel, as well as data on any health symptoms. The OEV-123 protocol was approved by the Ethics Committee of the Helsinki University Hospital (HUS/2231/2016) and Finnish Medicines Agency (FIMEA) and was recorded in the ClinicalTrials.gov database with the identifier NCT03729219. All subjects provided their informed, written consent. This ethical assessment was for OEV-123.

### 2.4. Statistics

Our objective in selecting a statistical approach was to strive to achieve both the efficient use of limited available data and the acknowledgement of between-individual and between-item differences, while also the modeling of missing data. Multilevel models efficiently pool information across different levels of data (individuals, items, measurements) and can also flexibly model item responses and missing data in a Bayesian statistical setting. Therefore, we performed Bayesian item response modeling with the brms R package [15,16], estimating the models with the Stan software implementation for Hamiltonian Monte Carlo sampling [17]. In essence, our model is a multilevel regression equivalent of the Graded Response Model of item response theory [18]. It is straightforward to regress the latent mental distress assessed by GHQ-12 items on covariates in the Bayesian multilevel formulation of the item response model. The latent continuous-valued mental distress becomes a function of the linear prediction equation:

$$\eta_{\mathrm{pi}}={\;\theta }_{p}+\overset{J}{\underset{j=1}{\sum }}\beta_{j}x_{\mathrm{jpi}},$$

where p denotes index persons, i denotes items, θ_p_ denotes person-specific mental distress, and x denotes the J selected, possibly person- and item-specific, covariate variables with estimable regression coefficients β. In addition to the linear prediction equation, the modeled item responses depend on a cumulative logit link function, estimable item discrimination, and item threshold parameters. The threshold parameters link the modeled continuous-valued latent responses with the observed ordinal-valued GHQ-12 item categories. The thresholds can be fully item-specific (3 × 12 parameters) or modeled via intercepts shared by items, as well as an item-level random effect and its correlation with the discrimination random-effect (3 + 2 parameters). The more heavily parameterized model is considered more “complex”.

A model can be considered extremely simple if it is inferior to a more complex model in terms of the leave-one-out cross-validation information criterion [19]. A model is considered extremely complex, unidentifiable or otherwise problematic if the Hamiltonian Markov chains estimation cannot be monitored to convergence (R^ < 1.05) or if it contains unnecessary features in terms of the aims. We started from a simple model and built complexity as needed, including missing data modeling. Although multilevel modeling efficiently uses all available data, including partial observation vectors for GHQ-12, we used multiple imputations with chained equations and a predictive mean matching method to investigate any potential attrition bias [20,21]. The Bayesian multiple imputation was applied to 100 imputed datasets using the brms and mice R packages, as previously recommended [22]. In addition to the Bayesian item response models, basic statistics and linear regression models were used to characterize the data. In regression modeling, cyclic patterns, such as time of year, can be modeled with cosine- and sine-transformed time covariates, which we used as well [23].

## 3. Results

### 3.1. Sample Characteristics

Table 1 shows a few basic sample characteristics. Up to 28% attrition is evident in the item responses toward the last data collection wave. The attrition mostly represents individual-level missing data: Only two participants reported some, but not all GHQ-12 items in waves 0 and 2, and none in wave 1. Nevertheless, our imputation approach in the next section deals with both types of missing data.

GHQ-12 sum scores in Table 1 suggest that the mental distress was lowest in the middle wave, as hypothesized. The difference among the waves in GHQ-12 was statistically significant in a complete-data linear model (F = 11.61, numerator d.f. = 2, denominator d.f. = 425, *p* < 0.001). Specifically, the middle wave (wave 1) sum score differed from the wave 0 (*p* < 0.001) and wave 2 (*p* = 0.008) sum scores in paired samples t-tests, whereas, waves 0 and 2 did not statistically significantly differ from each other (*p* = 0.072). Then, we investigated the more involved statistical approaches for testing our hypothesis and sum score estimates.

### 3.2. Multilevel Item-Response Modeling

In a Bayesian multilevel model, we fit the random-effect and shared intercepts for ordinal thresholds. We found that wave 1 indeed implicated lower levels of mental distress compared to waves 0 and 2 (Model 1 in Table 2). The estimates remained similar in a multiple imputation version of the model (Model 2 in Table 2), suggesting that the effects of the trip on the latent response was robust to attrition bias. However, the leave-one-out cross-validation information criterion of Model 1 was inferior to Model 3 with item-specific threshold estimation (∆LOOIC = −132.7); although the effects of the measurement wave were slightly attenuated in Model 3, they remained significant and aligned with our hypothesis (Model 3 vs. 1 in Table 2). Finally, we further adjusted for the time of year for the trip and for getting diarrhoea during the trip and/or after it. However, these adjustments had a negligible effect on the hypothesis pertaining to the effects of the trip (Model 4 in Table 2).

**Figure 1:**
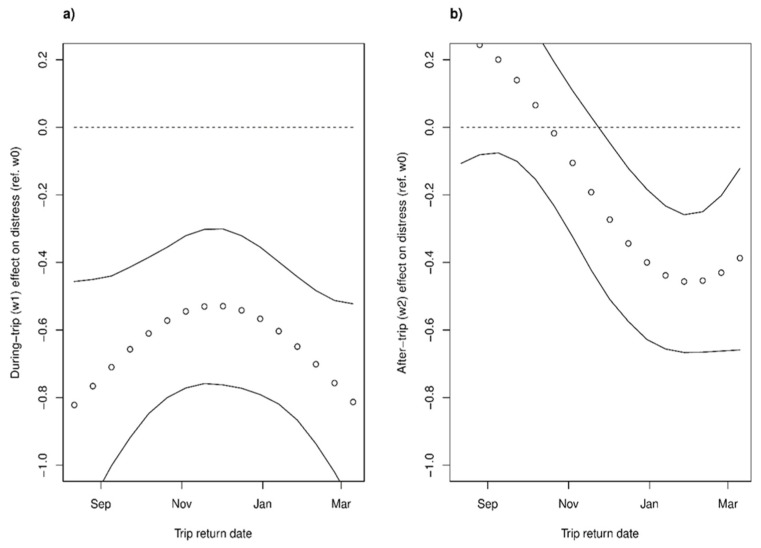
Posterior effects and 95% credible intervals by time of trip return ^1^. ^1^ Circles show, for each unique trip-return date in the data, posterior means for the sum of the main-effect coefficient as well as the multiplicative interaction-effect on the date. Solid lines show the 95% credible intervals. A dashed line is drawn at no effect for illustrative purposes. (**a**) Wave 1, or during-trip, regression coefficient. (**b**) Wave 2, or after-trip, regression coefficient.

Furthermore, we investigated interactions between the trip and the time-of-year and observed a significant interaction between the wave 2 effect and the cosine term of the model. This result is possibly best illustrated by plotting how the time of the trip influenced the total coefficient of each study wave: Trips taken after November led to similar, but more lasting decreases in mental distress compared to pre-November trips (Figure 1). Having diarrhoea after the trip increased mental distress, but diarrhoea during the trip did not (Model 4 in Table 2).

### 3.3. Multilevel Model Predictions in Units of the GHQ-12 Score

Regarding the average GHQ-12 score per study wave, the Bayesian item response models yielded very similar posterior predictions compared to the observed averages in the raw data (Table 3). These data both provide meaningful units for readers familiar with GHQ-12 and serve as a posterior predictive check that our model inferences appear robust and reasonable. Irrespective of the model, we found similar levels of support for our original hypothesis when examined in units of the total GHQ-12 score. While some parameter estimates slightly shifted in imputation (Model 1 vs. 2 in Table 2), the shifts appear to cancel out when predicting the total GHQ-12 score (Model 1 vs. 2 in Table 3). Modeling item-specific thresholds had little effect on the average GHQ-12, but appeared to better capture variation in GHQ-12, leading to better estimates of the standard deviation (Model 3 in Table 3).

## 4. Discussion

Despite the common view that taking a vacation has a positive psychological impact, studies exploring the impact of a vacation on well-being are scarce. This prospective observational study assessed the psychological well-being of travelers visiting a tropical region, Benin in West Africa. We found that a tropical holiday increases psychological well-being, with no gender- or age-specific differences. The beneficial effect diminished over 1 month, although it did not fully settle back to the pre-vacation status. This timeline is comparable to a study by Kuhnel (2011) on the effects of a holiday on burnout symptoms, in general [24]. In fact, even short vacations may alleviate stress, but for a shorter period [1]. In this study, winter vacations yielded long-lasting psychological benefits, which may be related to the fact that the participants originated from a Nordic country with long and dark winters. Accordingly, we observed that time of year moderated the longevity of the vacation’s positive mental-health effects. The results support our initial hypothesis that a tropical vacation alleviates psychological distress and the results withstood multiple imputations and Bayesian multilevel item response modeling, remaining largely unchanged.

To the best of our knowledge, this is the first study to assess the impact of carefully assessed TD on psychological well-being: Conducting the present study parallel to the OEV-123 field trial allowed us to obtain the data of the TD rates as evaluated in the field trial. As many as 71% of the participants of the present study reported TD, some of them experiencing the symptoms while abroad, and others after return. The TD incidence rates in this study are in line with the TD rate of 69%, which was reported in an earlier prospective study of 460 international travelers [25]. Interestingly, in the present study, TD experienced abroad did not affect psychological well-being, while TD after return decreased it. A study from the Netherlands assessed inconvenience caused by TD during subtropical trips [26]. Irrespective of TD severity, even the participants who were forced to alter their planned activities or to stay indoors tended to consider TD less of a problem upon return than they had thought it would be before departure [26]. An earlier 1999 study conducted in Jamaica reported that TD affected quality of life and general well-being negatively, suggesting travelers would benefit from the ETEC vaccine [27].

Sirgy et al. [28] have assessed specific sources for positive and negative effects with significant impact on tourists’ overall sense of well-being. Their findings support the notion that reducing the incidence of a negative effect in the health domain is more important to health well-being than inducing a positive effect. Therefore, in relation to tourism, tourist operators should develop programs and services to ensure that tourists do not get tired and exhausted while touring, do not get sick, do not gain weight, and feel protected from crime [28]. To increase well-being, the authors advised on programs that allow tourists to feel that they are breaking away from their daily routines, to experience new places and stay outdoors, and to enjoy the travel and lodging accommodations—all these experiences were provided to the participants of this study.

Although the present study was conducted among volunteers participating concomitantly in an RCT study, we believe that the RCT study did not have a major impact on our results. This is due to the fact that the RCT study had been planned to make the travel resemble a regular holiday as closely as possible. Therefore, the program only included a minimal number of contacts between its participants and study personnel. Indeed, apart from the initial lecture, during their 12-day stay, healthy participants only had three official contacts with the RCT personnel, each with a duration of less than 30 min. Those with TD or other health problems were asked to be in contact at the beginning of each episode. The only other restriction for the participants was that they had to be accommodated in selected hotels and not to make overnight visits elsewhere. By contrast, they were allowed to participate in various daytrips and any local activities. In fact, voluntary cultural excursions were organized for them by the Finnish African Cultural center Villa Karo, located in the same fishing village. The idea was to provide the participants a chance to get to see Africa off the beaten track. They were allowed to choose their daily activities and have their meals wherever they wanted. As a result, with these minimal restrictions, they were considered to represent regular holiday travelers.

### Limitations and Strengths

The limitations of this study include some missing information, which is often unavoidable in studies using self-rating questionnaires. Multiple imputations were used in order that we could report all the available information and partly correct for possible attrition-related bias in estimates, as described in the Methods Section. This is an observational longitudinal study by design. Moreover, it is possible that participants with concerns of their well-being chose to enroll in this study on psychological well-being and this might explain the moderately high initial distress percentage, which decreased during participation in the study to approximately the level of the general population sample. Namely, psychological distress and symptoms were reported by 12% of Finnish adults in a large population sample in 2018 and by 14% in 2020 [12,29]. In the present study, 24% of the participants reported psychological distress before the vacation as compared to 16% after the vacation, implying that participants probably represented, in general, healthy, but initially psychologically distressed adults.

The strengths of this study include the use of the validated questionnaire as well as the homogeneity of the study population, which allowed us to avoid some major confounding factors related to studies exploring the impact of vacations. All of the participants visited the same destination and stayed at the same hotels, resulting in fairly stable external conditions throughout the study.

Among business travelers, international business travel was significantly associated with a lower body mass index, lower blood pressure, excess alcohol consumption, sleep deprivation, and a reduced confidence in working ability compared to the zero international trip group [30]. While vacation travelers have been less well studied, clearly time-zone differences are one potentially challenging factor for both groups. The time-zone between Finland and Benin differs only by 1 or 2 h (depending on transitions to summer/wintertime in Finland). Therefore, we did not have to consider jet lag as a confounding factor.

## 5. Conclusions

Our data confirm the results of previous studies by showing that a holiday in the tropics supports psychological well-being. Traveling to a tropical country from a Nordic country in wintertime yielded longer-lasting well-being benefits than summertime travels. In addition, our study adds to the scarce research on the impact of TD on well-being. Although the risk of TD among visitors to these regions is substantial, the impact of TD appeared to depend on its timing: TD during the trip did not have an impact on the reported well-being, while TD after the return decreased it.

## Figures and Tables

**Table 1:** Characteristics of study participants and psychological distress (GHQ-12 scores) during the study waves.

	Count	Percentage	
N (Total sample size)	174	-	-
Sex 1 (Men)	47	27%	-
N_{wave 0 item responses}_	2038	98%	-
N_{wave 1 item responses}_	1596	76%	-
N_{wave 2 item responses}_	1498	72%	-
Diarrhoea during trip	124	71%	-
Diarrhoea after trip	22	13%	-
Wave 0 symptoms (score ≥ 3)	40/170	24%	-
Wave 1 symptoms (score ≥ 3)	13/133	10%	-
Wave 2 symptoms (score ≥ 3)	20/125	16%	-
	**Mean**	**S.d.**	**Range**
Age	40.16	16.49	18–65
Average wave 0 GHQ-12 sum	10.52	4.30	0–36
Average wave 1 GHQ-12 sum	8.28	3.58	0–36
Average wave 2 GHQ-12 sum	9.55	4.09	0–36
Wave 0 GHQ-12 interp. score	1.64	2.49	0–12
Wave 1 GHQ-12 interp. score	0.66	1.38	0–12
Wave 2 GHQ-12 interp. score	1.06	2.04	0–12

**Table 2:** Bayesian multilevel item response model posterior means (β) and 95% credible intervals (CI).

	Model 1		Model 2		Model 3		Model 4	
Fixed effect ^1^	*β*	CI	*Β*	CI	*β*	CI	*β*	CI
Threshold [1]	−1.54	(−2.45, −0.73)	−1.79	(−2.81, −0.86)	-	-	-	-
Threshold [2]	2.73	(1.81, 3.65)	3.24	(2.21, 4.27)	-	-	-	-
Threshold [3]	6.23	(5.06, 7.50)	7.35	(5.97, 8.83)	-	-	-	-
disc_Intercept	−0.06	(−0.30, 0.16)	−0.29	(−0.51, −0.07)	0.08	(−0.18, 0.33)	0.06	(−0.19, 0.30)
sex 2 (ref. 1)	−0.03	(−0.41, 0.34)	0.08	(−0.31, 0.46)	−0.15	(−0.50, 0.22)	−0.06	(−0.41, 0.28)
z (age)	−0.02	(−0.18, 0.13)	−0.04	(−0.21, 0.13)	−0.04	(−0.20, 0.12)	−0.05	(−0.22, 0.12)
wave 1 (ref. 0)	−0.76	(−0.95, −0.57)	−0.97	(−1.22, −0.74)	−0.60	(−0.76, −0.46)	−0.63	(−0.79, −0.48)
wave 2 (ref. 0)	−0.32	(−0.49, −0.15)	−0.45	(−0.66, −0.25)	−0.21	(−0.35, −0.07)	−0.21	(−0.35, −0.07)
cos (t/360)	-	-	-	-	-	-	0.01	(−0.24, 0.24)
sin (t/360)	-	-	-	-	-	-	−0.18	(−0.54, 0.18)
diarrhoea, trip	-	-	-	-	-	-	−0.24	(−0.59, 0.11)
diarrhoea, after	-	-	-	-	-	-	0.53	(0.04, 1.03)
Random eff.	*σ*	CI	*Σ*	CI	*σ*	CI	*σ*	CI
sd (Threshold)	1.29	(0.81, 2.14)	1.51	(0.95, 2.45)	-	-	-	-
sd (disc)	0.32	(0.20, 0.54)	0.28	(0.18, 0.46)	0.39	(0.23, 0.64)	0.37	(0.22, 0.62)
cor (disc, thr)	0.35	(−0.23, 0.78)	0.45	(−0.12, 0.83)	-	-	-	-

^1^ Note: β refers to the posterior mean of a model parameter, which is the regression coefficient only in the case of fixed effects; “disc” refers to the item discrimination parameter, but on a log-scale, thus actual discrimination is always positive; Model 1 is with fixed thresholds as well as a random effect, Model 2 is the same with multiple imputations, while Model 3 uses item-specific threshold parameters, and Model 4 introduces the time-of-year and diarrhoea covariates to Model 3. In cos (t/360), t denotes the days since the end of the first trip in the data; i.e., days since 12 August 2018. The data contained 16 unique trip endings, the last one being 10 March 2019. The functions z, cos, sin, sd, and cor refer to the z-score, cosine, and sine transformations, standard deviation, and correlation, respectively.

**Table 3:** Posterior predictive checks on the observed and predicted average GHQ-12 sum score (range 0–36) per study wave ^1^.

	Raw Data		Model 1		Model 2		Model 3	
	Mean	SD	Mean	SD	Mean	SD	Mean	SD
Wave 0	10.52	4.30	10.37	3.66	10.40	3.36	10.38	4.07
Wave 1	8.28	3.58	8.36	3.45	8.34	3.24	8.44	4.06
Wave 2	9.55	4.09	9.53	3.70	9.44	3.31	9.65	4.23

^1^ Note: The values are the observed and predicted average GHQ-12 sum scores and the GHQ-12 standard deviations per study wave. Models 1–3 correspond to those in Table 2.

## Data Availability

Please contact the corresponding author for the original dataset.
